# FGF family members differentially regulate maturation and proliferation of stem cell-derived astrocytes

**DOI:** 10.1038/s41598-019-46110-1

**Published:** 2019-07-03

**Authors:** Ekaterina Savchenko, Gabriel N. Teku, Antonio Boza-Serrano, Kaspar Russ, Manon Berns, Tomas Deierborg, Nuno J. Lamas, Hynek Wichterle, Jeffrey Rothstein, Christopher E. Henderson, Mauno Vihinen, Laurent Roybon

**Affiliations:** 1 Department of Experimental Medical Science, BMC D10, Faculty of Medicine, Lund University, SE-22184 Lund, Sweden; 2 MultiPark and Lund Stem Cell Center, Faculty of Medicine, Lund University, SE-22184 Lund, Sweden; 3 Department of Experimental Medical Science, Faculty of Medicine, BMC B13, Lund University, SE-22184 Lund, Sweden; 4 Department of Experimental Medical Science, Faculty of Medicine, BMC B11, Lund University, SE-22184 Lund, Sweden; 5 Life and Health Sciences Research Institute (ICVS), School of Medicine, University of Minho, Braga, Portugal, and ICVS/3B’s, PT Government Associate Laboratory, Braga/Guimarães, Portugal; 6 Anatomic Pathology Service, Pathology Department, Hospital and University Center of Porto, Largo Professor Abel Salazar, 4099-001 Porto, Portugal; 7 Center for Motor Neuron Biology and Disease, Columbia Stem Cell Initiative, Columbia Translational Neuroscience Initiative, Columbia University, New York, NY 10032 USA; 8 Department of Pathology and Cell Biology, Neurology, and Neuroscience, College of Physicians and Surgeons, Columbia University, New York, NY 10032 USA; 9 Project A.L.S./Jenifer Estess Laboratory for Stem Cell Research, New York, NY 10032 USA; 10 Solomon H. Snyder Department of Neuroscience, Johns Hopkins University School of Medicine, Baltimore, MD 21205 USA; 11 Brain Science Institute, Johns Hopkins University School of Medicine, Baltimore, MD 21205 USA; 12 Department of Neurology, Johns Hopkins University School of Medicine, Baltimore, MD 21205 USA; 13 Program in Cellular and Molecular Medicine, Johns Hopkins University School of Medicine, Baltimore, MD 21205 USA; 14 Department of Rehabilitation and Regenerative Medicine, College of Physicians and Surgeons, Columbia University, New York, NY 10032 USA; 15 Target ALS Foundation, New York, NY 10032 USA; 16 Present Address: Biogen Inc., Cambridge, MA 02142 USA

**Keywords:** Glial stem cells, Stem-cell differentiation

## Abstract

The glutamate transporter 1 (GLT1) is upregulated during astrocyte development and maturation *in vivo* and is vital for astrocyte function. Yet it is expressed at low levels by most cultured astrocytes. We previously showed that maturation of human and mouse stem cell-derived astrocytes – including functional glutamate uptake – could be enhanced by fibroblast growth factor (FGF)1 or FGF2. Here, we examined the specificity and mechanism of action of FGF2 and other FGF family members, as well as neurotrophic and differentiation factors, on mouse embryonic stem cell-derived astrocytes. We found that some FGFs – including FGF2, strongly increased GLT1 expression and enhanced astrocyte proliferation, while others (FGF16 and FGF18) mainly affected maturation. Interestingly, BMP4 increased astrocytic GFAP expression, and BMP4-treated astrocytes failed to promote the survival of motor neurons *in vitro*. Whole transcriptome analysis showed that FGF2 treatment regulated multiple genes linked to cell division, and that the mRNA encoding GLT1 was one of the most strongly upregulated of all astrocyte canonical markers. Since GLT1 is expressed at reduced levels in many neurodegenerative diseases, activation of this pathway is of potential therapeutic interest. Furthermore, treatment with FGFs provides a robust means for expansion of functionally mature stem cell-derived astrocytes for preclinical investigation.

## Introduction

Astrocytes, the most common cell type in the brain, play a crucial role in the maintenance of homeostasis of the central nervous system (CNS)^1^. They act as structural and trophic support for nervous system, regulate cerebral blood flow, provide energy substrate for neurons, and are involved in the formation and modulation of synapses^2–5^. Under pathological stimuli, activated astrocytes, which are characterized by the up-regulation of glial fibrillary acidic protein (GFAP) and altered morphology, can play a key role in detrimental neuro-inflammatory processes^1,6^.

Another important function of astrocytes is the uptake of the main excitatory neurotransmitter glutamate^7,8^, by high affinity transporters glutamate aspartate transporter (GLAST; known as EAAT1 in human and encoded by the Slc1a3 gene) and glutamate transporter 1 (GLT1; known as EAAT2 in human and encoded by the Slc1a2 gene)^9^. Loss of either transporter produces a tonic increase in extracellular glutamate concentration^9^, which can lead to excitotoxicity via excessive activation of glutamate receptors and result in oxidative stress and neuronal cell death^8,10,11^. Reduced GLT1 protein levels have been observed in patients diagnosed with Huntington’s Disease (HD) or Amyotrophic Lateral Sclerosis (ALS), as well as in several animal models of these NDDs^12–16^. In fact, reduced GLT1 levels in astrocytes have been associated with other neurological disorders, such as Alzheimer’s disease (AD), ischemic stroke, and epilepsy^14,17,18^. Therefore, targeting GLT1 expression may be an interesting therapeutic approach in these disorders.

The expression of GLT1 is associated with maturation of astrocytes^19^ and is dependent upon soluble factors secreted by neurons^10,20^. FGF 1 and 2 (also known as acidic FGF and basic FGF, respectively) are soluble proteins mainly secreted by neurons that contribute to the maturation of astrocytes^21–23^. Increased extracellular glutamate levels, which can be caused by reduced expression of GLT1^9^, significantly enhance the release of FGF2 from cortical neurons^24^. FGFs may be homeostatic regulators of GLT1 expression. However, nothing is known about the regulation of GLT1 by other FGF subfamily members and other soluble factors, including ciliary neurotrophic factor (CNTF) and bone morphogenetic protein 4 (BMP4), which are commonly employed to generate astrocytes *in vitro*.

The FGF family contains 23 members and can be divided into seven subfamilies^25–27^. In general, FGFs act as paracrine factors that bind to the FGF receptor in the presence of the co-factor heparin or heparin sulphate proteoglycans. FGFs regulate cell proliferation, differentiation, and survival^22,23,27,28^ and have opposing roles in inflammation regulation^29–31^. Thus, FGFs have various functions and they may differently regulate GLT1.

In this report, we aimed to clarify the function of various members of the FGF family on maturation of astrocytes. First, we generated mouse ESC (mESC)-derived spinal cord astrocytes and examined their transcriptome prior to and upon FGF2 treatment. Next, using an innovative screening assay employing whole culture well imaging, we examined whether other factors, including FGF family members with paracrine activities, could upregulate GLT1 expression. Additionally, we examined the transcriptome of FGF18-treated astrocytes, which like FGF2-treated astrocytes, upregulated GLT1 expression without strong effect on astrocyte proliferation. Finally, we examined the A1/A2 phenotype of the FGF2- and FGF18-treated astrocytes^32^, and found that FGF2- and FGF18-treated astrocytes were not toxic to motor neurons (MNs), as opposed to BMP4-treated astrocytes. Our study showed that FGF family members differentially regulate maturation of stem cell-derived astrocytes. We identified several FGFs as potential therapeutic targets in diseases where loss of GLT1 prevails.

## Results

### FGF2 is a strong inducer of astrocytic GLT1 and growth

We employed a previously established protocol to generate astrocytes from mESC^19^. First, mESC were differentiated into MN cultures for 7 days using caudalizing retinoic acid (RA) and ventralizing smoothened agonist (SAG) agents^33^. The robustness of the MN differentiation step was assessed by visualizing MNs using a *Hb9::GFP* reporter line (Fig. S1). The MN cultures, which contained neural progenitors, were exposed to epidermal growth factor (EGF) and FGF2 allowing the neural progenitors to expand as free-floating neurospheres for two weeks with an intermediate step of generation of secondary neurospheres. On day 21, neurospheres, which were devoid of *Hb9::GFP* MNs and mainly contained neural progenitors, were mechanically dissociated and progenitors were differentiated into astrocytes on adherent surfaces using medium supplemented with 10% FBS for 2 weeks. On day 35 of differentiation, cultures were enriched in immature astrocytes and devoid of cells positive for neuronal marker MAP2 or oligodendrocyte marker CNPase^19^.

**Figure 1:**
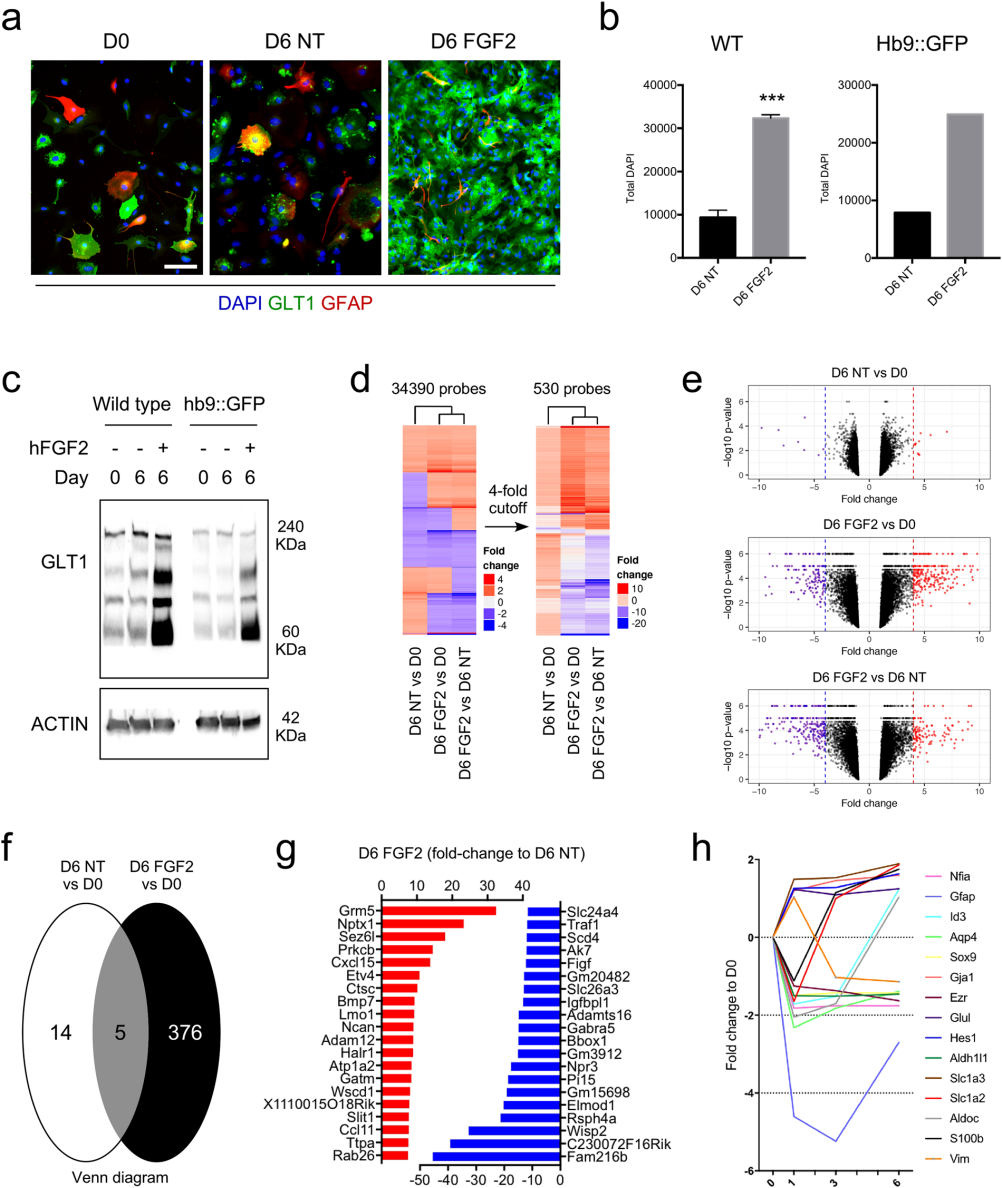
Increased abundance of GLT1-expressing astrocytes upon FGF2 treatment. (**a**) FGF2 treatment robustly increases GLT1 levels in astrocyte cultures generated from mESC lines. Scale bar = 50 μm. (**b**) FGF2 treatment robustly increases astrocyte growth. A 3-fold increase in number of astrocytes was identified in cultures treated with FGF2 when compared to non-treated ones (mean ± SEM; n = 3 independent differentiations for the WT mESC line, and n = 1 for the *Hb9::GFP* line). *P* value: ***P < 0.001. Scale bar = 50 μm. (**c**) Representative Western blot of mESC-derived astrocyte cultures prior to and following treatment or not with FGF2. GLT1 protein is abundant in cultures treated for 6 days with FGF2, compared to non-treated astrocyte cultures of the same age and cultures prior to treatment. Actin protein was used as loading control (blots are representative of two independent experiments). (**d**) Heat map profiles for gene expression. The panel to the left shows all genes, while that to the right are genes with at least four-fold difference up or down. (**e**) Volcano plots of fold change of gene expression vs -log of p-value. The dashed lines show expression change four-fold up and down. Genes with expression change >4-fold are colored red, those expressed <−4 fold in blue, while those <4 and >−4 are colored black. P-values close to zero where replaced by 10^−6^. (**f**) Venn diagram showing overlap of genes with 4-fold expression change between the D6 NT vs D0 and D6 FGF2 vs D0. (**g**) List of top 20 genes up (red bars) and down (blue bars) regulated with FGF2 treatment. (**h**) Fold expression changes of canonical astrocytic markers, in cultures treated with FGF2 for 1, 3 and 6 days. Data are normalized to D0.

The astrocytes were then used to verify our previous findings of FGF2-induced GLT1 expression in astrocytes^19^. First, the astrocyte cultures were treated with FGF2 (50 ng/mL), which led to a robust expression of astrocytic GLT1, as measured by immunocytochemistry (Fig. 1a). FGF2 also stimulated astrocyte growth by 3-fold (Fig. 1b). We further confirmed the increase in GLT1 level in astrocyte cultures with Western blotting (Fig. 1c). These changes were uniform for astrocyte cultures generated from two different mESC lines, the *Hb9::GFP* reporter line and a newly derived wildtype (WT) line (Fig. 1b,c).

Whole transcriptome analysis was performed to understand the effects of FGF2 treatment in astrocytes (Table S1). The samples analyzed were: (i) non-treated astrocytes at day zero (D0), (ii) non-treated astrocytes aged for 6 days (D6 NT) in culture, and (iii) FGF2-treated astrocytes aged for 6 days (D6 FGF2) in culture. The gene expression levels were compared for the 3 conditions (Fig. 1d,e). Since astrocytes generated from the *Hb9::GFP* and WT mESC lines reacted similarly to FGF2 treatment, measured by increased GLT1 (Fig. 1c), we analyzed four samples per condition of which three where from astrocyte cultures generated from WT mESC line and one from the *Hb9::GFP* mESC line. 530 genes showed a 4-fold or higher change up or down (Fig. 1d,e). 376 genes were up and down regulated following FGF2 treatment, of which 5 were common to D6 NT and D6 FGF2 samples (Fig. 1f). Among the upregulated genes were the glutamate metabotropic receptor 5 (Grm5), which is important for calcium signaling^34^, and Slit guidance ligand 1 (Slit1) expressed in ventral spinal cord astrocytes^35^ (Fig. 1g). Interestingly, FGF2 had a minor effect on the up and down-regulation of common astrocytic genes (Fig. 1h). However, FGF2 triggered a significant over 2-fold up-regulation of Slc1a2 gene encoding for GLT1 (Fig. 1h).

**Figure 2:**
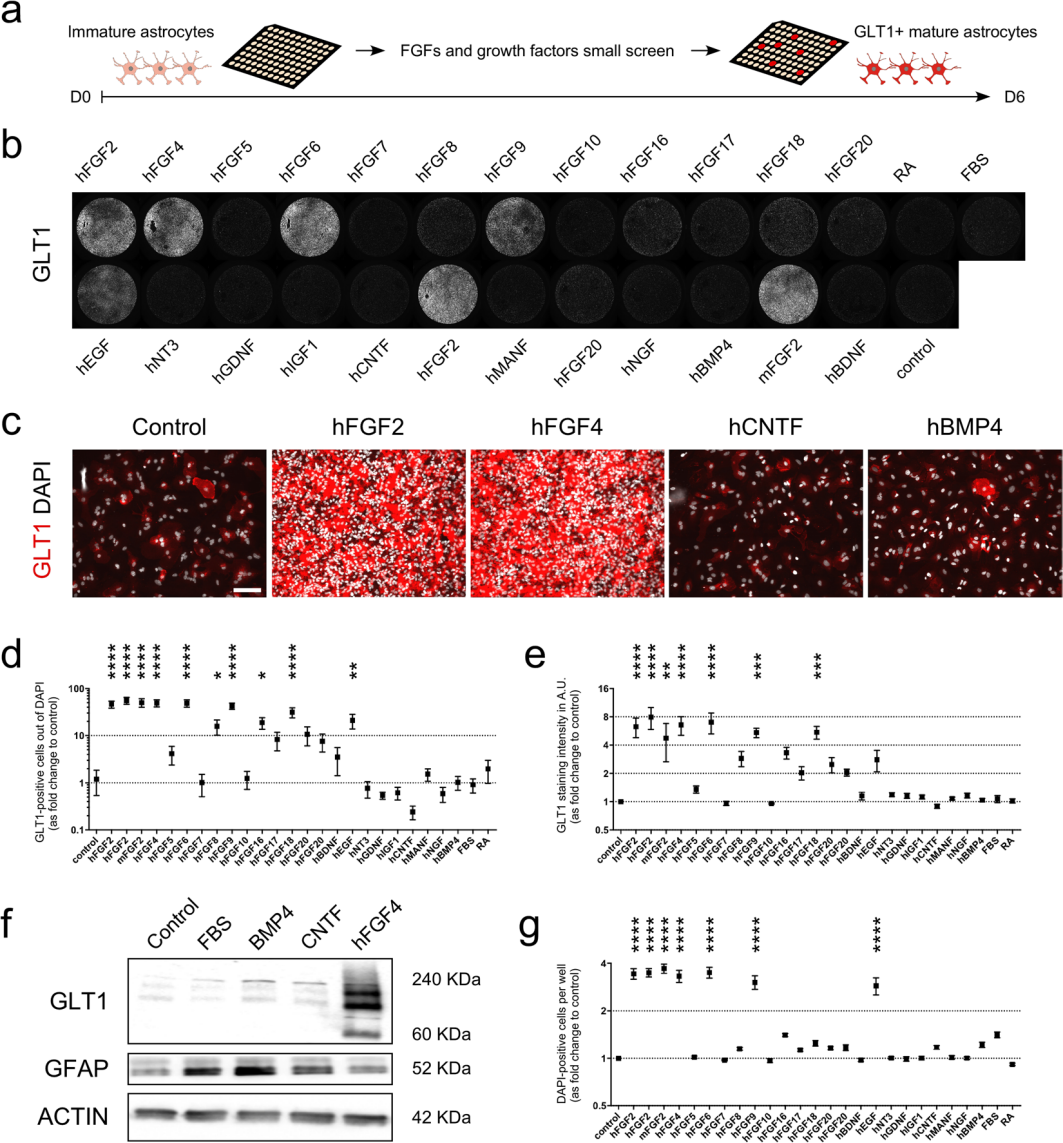
Identification of factors promoting astrocyte GLT1 expression and growth. (**a**) Overview of the screening workflow employed to examine astrocytic GLT1 up-regulation. (**b**) Whole well image depicting GLT1 immunofluorescence following immunocytochemistry. Representative images from one of the 4 screens are presented. Cultures were treated for 6 days with the conditions, with no media change. (**c**) High magnification images of non-treated, FGF4 and BMP4 treated astrocyte cultures, stained for GLT1 and DAPI (images are representative of n = 4 experiments). Scale bars = 200 μm (upper panel) and 50 μm (lower panel). (**d**) Bar diagram represents the proportion of GLT1-positive cells in each culture condition (data are presented as fold-change of the non-treated cultures). Mean ± SEM; treatment performed for n = 4 independent differentiations. *P* values: *P < 0.05; **P < 0.01; ***P < 0.001; ****P < 0.0001. (**e**) Bar diagram represents the average intensity staining per cell in each culture condition (data are presented as fold-change of the non-treated condition). Mean ± SEM; n = 4 independent differentiations. *P* values: *P < 0.05; **P < 0.01; ***P < 0.001; ****P < 0.0001. (**f**) Western blot analysis confirms the increased GLT1 levels observed using immunocytochemistry. GFAP, marker of reactivity and AQP4, canonical marker of astrocytes, were downregulated when GLT1 was upregulated in response to FGF4 treatment. Actin was used as housekeeping marker. 20 μg of proteins were loaded for each condition. (**g**) Bar diagram represents the total number of cells in each culture condition (presented as log_2_-fold change of the non-treated condition). Mean ± SEM; n = 4 independent differentiations. *P* values: *P < 0.05; **P < 0.01; ***P < 0.001; ****P < 0.0001.

Gene Ontology^36^ annotation enrichment was investigated using hypergeometric distribution. Significant enrichment was identified for GO term categories of upregulated genes (Fig. S2). They were enriched for regulation, metabolism, cellular compartment organization and response to stimuli. The molecular function terms include numerous categories associated with protein, nucleotide and ion binding. Of the cellular component terms, nucleus was the most altered followed by macromolecular complexes, membranes, and cytoskeleton. GO term enrichment also revealed categories associated with cell division machinery (Fig. S2), which confirmed the effect of FGF2 on astrocyte growth (Fig. 2b). No GO term category was significantly enriched among the downregulated genes. These data collectively show that FGF2 is a strong inducer of both astrocyte maturation and growth.

### Specificity of FGFs on astrocytic GLT1 expression and growth

**Figure 3:**
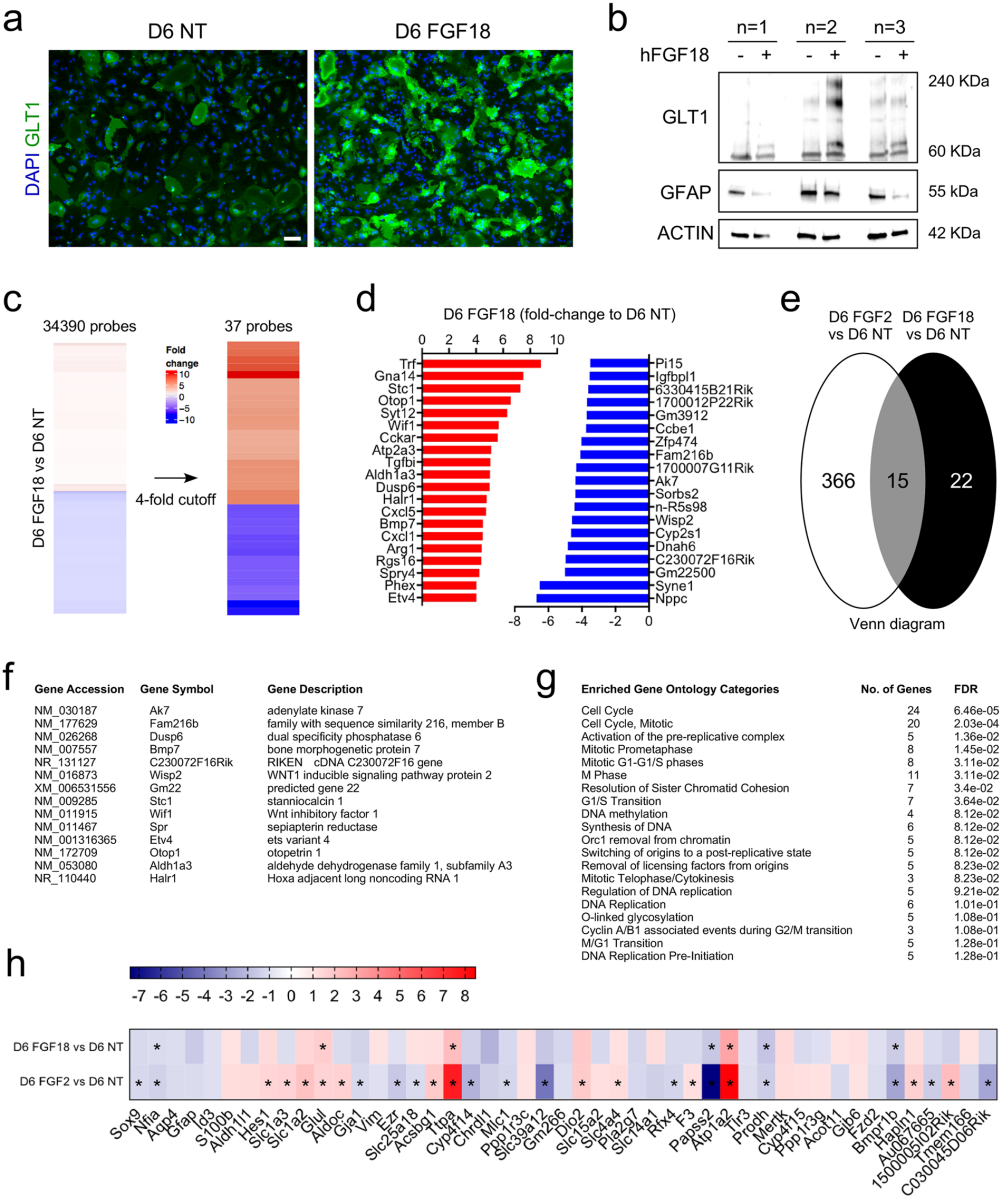
Effect of FGF18 on astrocyte cultures. (**a**) FGF18 treatment robustly increases GLT1 levels in astrocyte cultures. Images are representative of n = 3 independent experiments. Scale bar = 50 μm. (**b**) Western blot of mESC-derived astrocyte cultures prior to and following treatment or not with FGF18. GLT1 and GFAP are increased and decreased, respectively, in cultures treated for 6 days with FGF18, compared to non-treated astrocyte cultures of the same age. Actin protein was used as loading control (blots are representative of three independent experiments). (**c**) Heat map profiles for gene expression. The panel to the left shows all genes, while that to the right is for genes with at least four-fold difference up or down. (**d**) List of top 20 genes up (red bars) and down (blue bars) regulated with FGF18 treatment. (**e**) Venn diagram showing overlap of genes with 4-fold expression change between the D6 NT vs D0 and D6 FGF2 vs D0. (**f**) List of annotated genes common to FGF2- and FGF18-treated astrocyte cultures. (**g**) Significantly enriched GO Slim terms in the Reactome pathways for genes with over 1.5-fold expression change. (**h**) Heat map representing the expression of astrocyte-specific genes^54^ following treatment with FGF2 and FGF18 (blue and red colored squares show low and high expression, respectively, of genes of interest). Statistically significant changes are marked with an asterisk.

In human, there are 23 FGFs, which are grouped based on their sequence and evolutionary similarity into 7 subfamilies of which 5 have paracrine effects^37^. FGF2 belongs to the first family and we have previously showed that the two members of this family promote increased astrocytic GLT1 levels, with FGF2 having a greater efficacy compared to FGF1^19^. Since it was not known whether astrocytic GLT1 expression is restricted to members of the FGF1 subfamily, we screened for GLT1 inducers using immature mESC-derived astrocytes (Fig. 3a). We tested the effect of FGF4, FGF5, FGF6, FGF7, FGF8, FGF9, FGF10, FGF16, FGF17, FGF18 and FGF20, which represent the 4 other subfamilies with paracrine effect, namely FGF4, FGF7, FGF8 and FGF9 subfamilies (Table 1). We also included in our assay other factors that provide neurotrophic support (BDNF, NT3, GDNF, IGF1, MANF and NGF)^38–41^, and factors that participate in neural induction and differentiation, such as retinoic acid (RA)^42^ and FBS. CNTF and BMP4, soluble proteins known to promote mammalian astrocyte differentiation from neural progenitors^43,44^, whose effect on astrocytic GLT1 expression is unknown, were also tested. Prior to performing the screens on immature astrocytes, all factors were tested several times on mESC-derived *Hb9::GFP* MNs to evaluate their efficacy (Fig. S1). All the recombinant proteins were tested at a concentration of 50 ng/mL, and RA at 1 µM. Some factors were obtained from two different suppliers (FGF2 and FGF20); in the case of FGF2, both murine and human recombinant proteins were tested (Fig. 3b). We employed immunocytochemistry for GLT1 and DAPI staining as readouts for astrocyte maturation and growth. Interestingly, neither commonly employed factors known to exert neurotrophic support on MNs such as BDNF and GDNF (Fig. S1), nor BMP4 or CNTF, which are the gold standard factors routinely employed to generate astrocytes from pluripotent stem cells^45–48^, promoted GLT1 expression (Fig. 3b–g). BMP4, but not CNTF, increased the expression of GFAP, a marker of immature and reactive astrocytes, and aquaporin 4 (AQP4; Fig. S3). FGF2, FGF4, FGF6, FGF9, FGF16 and FGF18 led to a dramatic increase in both the number and intensity staining of astrocytes positive for GLT1, when compared to control cultures (Fig. 3b–e). We confirmed the effect of FBS, BMP4, CNTF and FGF4 with Western blotting (Fig. 3f). We identified FGF2, 4, 6 and 9, but not 16 and 18, as strong inducers of astrocyte proliferation (Fig. 3g).

**Table 1:** list of compounds employed in the study.

Factor	Final concentration	Provider
mFGF2	50 ng/mL	Thermo Fisher
hFGF2	50 ng/mL	R&D
hFGF2	50 ng/mL	PeproTech
hFGF4	50 ng/mL	PeproTech
hFGF5	50 ng/mL	PeproTech
hFGF6	50 ng/mL	PeproTech
hFGF7	50 ng/mL	PeproTech
hFGF8	50 ng/mL	PeproTech
hFGF9	50 ng/mL	PeproTech
hFGF10	50 ng/mL	PeproTech
hFGF16	50 ng/mL	PeproTech
hFGF17	50 ng/mL	PeproTech
hFGF18	50 ng/mL	PeproTech
hFGF20	50 ng/mL	PeproTech
hFGF20	50 ng/mL	R&D
hBDNF	50 ng/mL	R&D
hEGF	50 ng/mL	R&D
hNT3	50 ng/mL	R&D
hGDNF	50 ng/mL	R&D
hIGF1	50 ng/mL	R&D
hCNTF	50 ng/mL	R&D
hMANF	50 ng/mL	R&D
hNGF	50 ng/mL	R&D
hBMP4	50 ng/mL	Invitrogen
FBS	10%	Thermo Fisher
Retinoic acid	10 µM	Sigma

The data clearly demonstrate that astrocytes generated using standard growth factors (e.g. CNTF and BMP4) are not fully mature, and that the treatment of immature astrocytes with specific members of the FGF subfamily with paracrine activity robustly promoted their maturation, measured by increased number and proportion of GLT1-positive astrocytes per well, and increase GLT1 level as measured with Western blotting.

### Transcriptome analysis of FGF18-treated astrocytes

We next examined the transcriptional changes between dividing and non-dividing mature astrocytes. To this aim, we compared FGF2 and FGF18 since these two factors had a different effect on astrocyte growth, although both promoted GLT1 expression. In a new set of experiments, we confirmed GLT1 enhancement by immunocytochemistry and Western blotting, following FGF18 treatment (Fig. 3a,b). Images of stained cultures clearly revealed increased GLT1 intensity staining per cell in astrocyte cultures treated with FGF18 (Fig. 3a).

We next analyzed the transcriptome of FGF18-treated cultures (Table S2) and compared it to that of FGF2-treated ones. In total, 37 genes showed on average 4-fold or higher change up or down (Fig. 3c,d), and 15 probes (for which 14 genes were annotated) were common between D6 FGF2 and D6 FGF18 samples (Fig. 3e,f). Amongst upregulated genes common to FGF2 and FGF18 treatments was *Bmp7*, an abundant protein in adult mature astrocytes throughout the CNS^49^ and involved in the regulation of reactive gliosis^50,51^ and upregulated and neuroprotective in spinal cord injury^52,53^. GO term enrichment revealed that no categories were associated with cell division machinery when a 4-fold cut off was applied. However, some of these categories could be identified when utilizing 2-fold cut off (Fig. 3g), suggesting FGFs-induced GLT1 up-regulation to be associated with astrocytic growth, *in vitro*. When examining more specific astrocytic genes^54^, we observed a greater up-regulation of genes expressed by mature astrocytes^54^, with significant increase in *Slc1a2*, *Aldoc*, *Glul*, *Acsbg1*, *Ttpa*, *Dio2*, and *Atp1a2* mRNAs level; Fig. 3h) in FGF2- compared to FGF18-treated cultures. These data suggest that FGF2-treated astrocytes had matured more rapidly than FGF18-treated ones.

### BMP4-treated astrocytes fail to promote the survival of MNs

**Figure 4:**
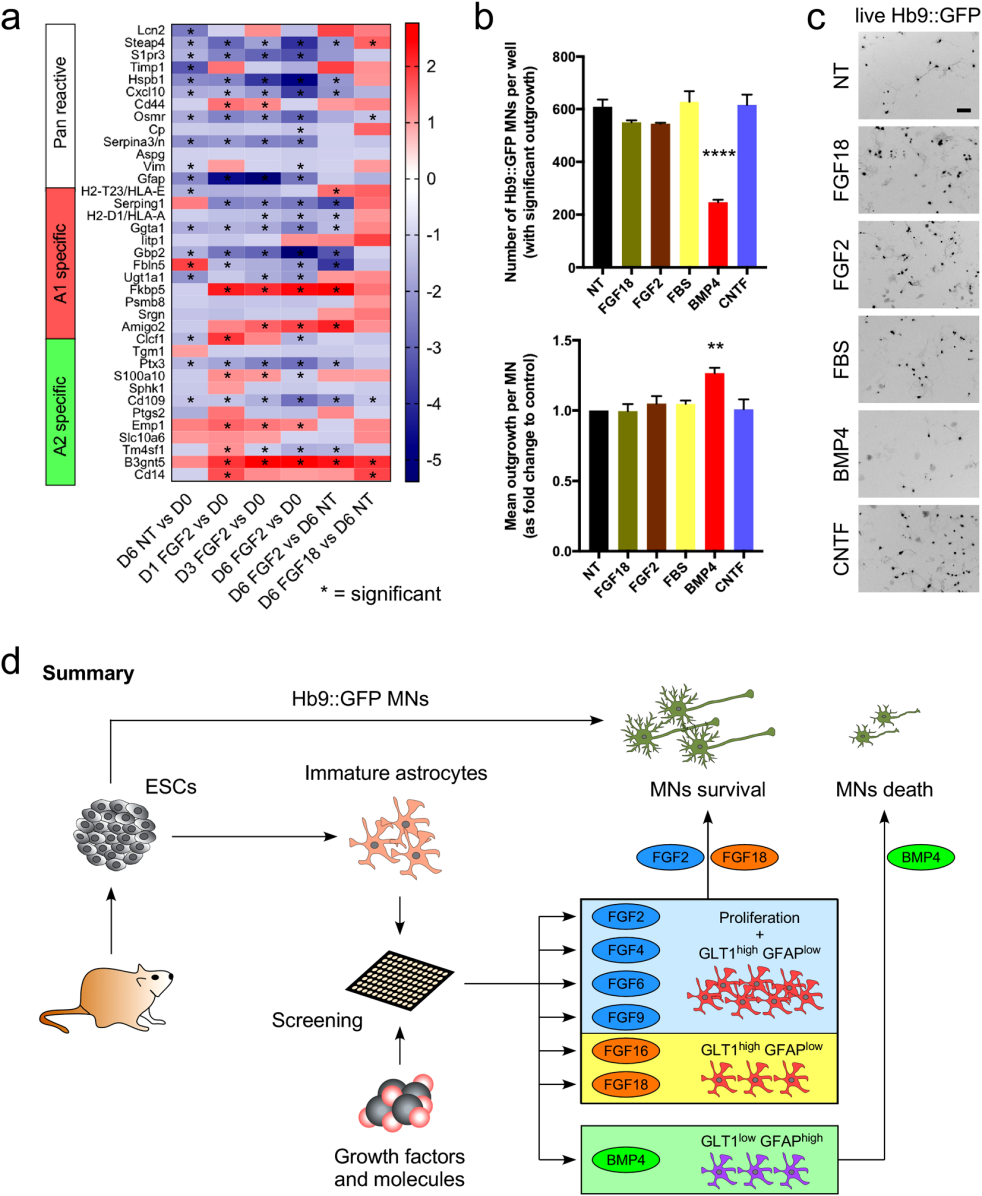
Effect of FGF18 on astrocyte cultures. (**a**) Heat map representing the expression of pan-reactive, A1- and A2-specific genes, for each analyzed condition. Data are represented as fold change up (red colored squares) or down (blue colored squares). Significant changes are marked with an asterisk. (**b**) Quantification of the total number of *Hb9::GFP* MNs and the mean outgrowth per MN after 2 days in co-culture with astrocytes treated with FGF18, FGF2, FBS, BMP4, CNTF or non-treated. Mean ± SEM; n = 3 independent experiments. P values: **P < 0.01; ****P < 0.0001. Scale bar = 50 μm. (**c**) Representative images of *Hb9::GFP* MNs after 2 days in co-culture with astrocytes treated with FGF18, FGF2, FBS, BMP4, CNTF and non-treated astrocytes. (**d**) Summary of our findings; FGF2, FGF4, FGF6, FGF9, FGF16 and FGF18 trigger GLT1 expression; FGF2, FGF4, FGF6 and FGF9 also promoted astrocyte growth. BMP4 triggers GFAP expression; BMP4-treated astrocytes failed to support the survival of MNs.

Astrocytes are heterogenous in the brain and *in vitro*. They can adopt different phenotypes, being mature or reactive pro-inflammatory when stimulated with FGF2 and TNF-alpha, respectively^19^ or become neurotoxic or neuroprotective^32^. To determine if FGF2 and/or FGF18 had directed the astrocytes towards an “A1 toxic” or an “A2 neuroprotective” phenotype, we examined up and down regulation of genes associated with these two phenotypes. Genes associated with the A1 toxic phenotype were mostly down-regulated by FGF2, although *Amigo-2* and *Fkbp5* were significantly upregulated, after 6 days of treatment (Fig. 4a). Interestingly, astrocytes exposed to FGF18 increased the expression of genes associated with the “A1 toxic” phenotypes, but the effect was not statistically significant (Fig. 4a).

Since neither an “A1 toxic” nor an “A2 neuroprotective” phenotype could be clearly identified for astrocytes treated with FGF18, while those treated with FGF2 showed mostly down-regulation of “A1 toxic” phenotype-related genes, we examined the effect of FGF2- and FGF18-treated astrocytes on MN survival. Astrocytes treated for 6 days with either FGF2 or FGF18 promoted the survival of *Hb9::GFP* MNs (Fig. 4b). This was also the case for non-treated astrocytes, and astrocytes treated with FBS or CNTF. Interestingly, astrocytes treated with BMP4, which is commonly used to generate astrocytes from stem cells and which promotes GFAP expression, failed to promote the survival of *Hb9::GFP* MNs (Fig. 4b).

Collectively, our data show that FGFs differentially regulate astrocyte maturation and growth, and that FGF2- and FGF18-treated astrocytes are not toxic to MNs, *in vitro*.

## Discussion

Maturation of astrocytes is associated with the increase and decrease of GLT1 and GFAP levels, respectively. Such phenotype can be induced *in vitro* by treatment of astrocytes with FGF1 family members FGF1 or FGF2^19^. Here, we confirmed these findings and further analyzed the mechanism of action of FGF2 in cultured astrocytes. FGF2 elicited different effects on the expression of canonical astrocytic genes, over time. While long-term treatment of astrocytes with FGF2 progressively increased levels of GLT1, short-time exposure led to a reduced expression of *Slc1a2* and several others astrocytic genes. Additionally, the expression of astrocyte canonical genes, *Slc1a3*, *Gja1* (encoding for connexin 43), *Glul* (encoding for glutamine synthetase) and *Id3* was induced while the expression of *Gfap*, *Vimentin* and *Aldh1l1* were strongly downregulated upon FGF2 treatment (Fig. 1h). These changes showing the differential expression of certain genes over time, may be consequent to the transcriptional adaptation of astrocytes to a new environment or an effect of the initiation of cell proliferation. GO enrichment analysis of the whole transcriptome revealed that most enriched genes were associated with intracellular processes and cell division. The increased proliferation of astrocytes has been observed in cell cultures treated with FGF2. Indeed, FGF2 is the most common growth supplement and is known to regulate proliferation, differentiation, migration, and survival of different cell types^55,56^.

FGF2 and FGF1 belong to the FGF1 subfamily; the effects of the other 7 FGF subfamily members in induction of astrocyte maturation was previously not known. Here, we screened for inducers of astrocyte maturation. The screen employed members of the other FGF subfamilies with paracrine effect and neurotrophic and differentiation factors, including BMP4 and CNTF, common inducers of astrocyte differentiation. We identified several additional FGF members that significantly enhanced astrocytic *Slc1a2* expression *in vitro*. Notably, FGF2, FGF4, FGF6 and FGF9 induced cell proliferation, whereas FGF16 and FGF18 did not. None of the other factors studied affected GLT1 level, although BMP4 elicited an increase in *Gfap* and *Aqp4* expression, in line with previous reports^57^. Among FGFs with reduced proliferative capacities, FGF18 showed a greater potential effect on astrocytes to induce GLT1 than FGF16. Therefore, we investigated the differences in gene expression patterns underlying the FGF18 treatment and compared them to those induced by FGF2 treatment. Similar to FGF2, FGF18 induced the upregulation of *Slc1a2* and downregulation of *Gfap*. Increased and decreased levels of EAAT2 and GFAP, respectively, and increase level of BMP7, common target of FGF2 and FGF18 (Fig. 3f), were also identified by Western blot following treatment of human astrocytes (Fig. S4 and data not shown). In addition, some other astrocyte-related genes^54^ were regulated in the same manner as FGF2-treated cells. Interestingly, the expression of Aldh1l1, a key enzyme for nucleotide biosynthesis and cell division^58^ that is selectively and very significantly decreased in mature spinal cord astrocytes through transcriptional inactivation of the *Aldh1l1* promoter^59^, elevated and downregulated following treatment with FGF2 and FGF18, respectively. Interestingly, *Slc1a3*, which characterizes immature differentiating cells confined to an astrocytic fate in the early postnatal brain^60^, was highly upregulated by FGF2. Hence, we cannot rule out the possibility that some of the observed changes in gene expression may be consequent, at least partly, for the enhanced proliferation induced by FGF2 treatment. Therefore, examining astrocyte response to treatment after several days is highly relevant to understand the long-term effect of the several compounds tested.

We also examined markers associated with an “A1 toxic” or an “A2 neuroprotective” phenotype^32^. FGF2 mostly decreased the expression of genes associated with an “A1 toxic” phenotype, and FGF2-treated astrocytes supported the survival of *hb9::GFP* MNs. This finding is in line with the recent use of FGF2 to protect both neurons and oligodendrocytes from the “A1 toxic” phenotype *in vitro*^32^. Interestingly, BMP4, a factor commonly employed to generate astrocytes from neural progenitors, rendered the astrocytes “toxic” to *hb9::GFP* MNs. Further analysis of the transcriptome of BMP4-treated astrocytes should indicate whether they can be categorized as “A1 toxic”.

FGF2 and FGF18 are secreted factors in the FGF1 and FGF8 subfamilies, respectively, that mediate signaling via FGF receptors. Although both ligands recognize the same receptors, but with different affinity, the ability of FGF2 to induce *Slc1a2* expression was more prominent. This may reflect different degrees of selectivity and activity of the FGF receptors for different FGFs. For example, FGF2 binds with a high affinity to FGFR1, while FGF18 preferentially binds to FGFR3^26,61^. In contrast to FGFR3, signaling through FGFR1 induces proliferation in both embryonic and human adult stem cells and is important for neurogenesis and proliferation and fate specification of radial glial cells in the cortex and hippocampus^62–65^. Our data also demonstrated that FGF2 treatment induced higher proliferation of astrocytes compared to FGF18 treatment.

Taken together, our data suggest that members of FGF family could enhance astrocytic GLT1 levels and prevent astrocyte reactivity indicated by the decrease of *Gfap* expression. Our findings are relevant to the elaboration of robust therapeutic strategies for the treatment of NDDs associated with impaired GLT1 function that could also be beneficial to MNs. For example, *in vivo* delivery of FGF18, loss of which in MNs leads to malformation of the neuromuscular junction^66^, could potentially delay MN die-back and prevent excitotoxic processes. Importantly, we introduced a new method to produce non-reactive/mature astrocytes to study astrocyte biology, development and maturation. Several protocols have been established to generate astrocytes from stem cells to model neurodegeneration, but most of them produce young, immature astrocytes that display low glutamate transporter levels^67,68^. Moreover, our approach provides a valuable platform for evaluation of the cross-talk between mature astrocytes and other brain cell types as well as for screening agents that could block astrocytic growth, which is characteristic for glioblastoma^69^.

## Experimental Procedures

### Ethics

All methods and experimental procedures were carried out in accordance with European and Swedish national rules and approved by Lund University. The derivation of mESC was approved by the ethical committee of Lund and Malmö (permissions #M43-15 and #M69-16).

### Derivation of mouse embryonic stem cell and maintenance

Wildtype mESC (C57BL/6 background) cell line was derived as previously described (Chumarina *et al*., 2017). Derivation of the *Hb9::GFP* mESC line has been reported (Wichterle *et al*., 2002). Mouse ESCs (mESCs) were maintained on a monolayer of irradiated CF1 feeders (Globalstem), in mESC culture medium composed of DMEM medium, 15% fetal bovine serum (FBS), penicillin/streptomycin (P/S; 100 Units/mL and 100 mg/mL, respectively), 2 mM L-glutamine (GIBCO), 100 mM non-essential amino acids (NEAA, GIBCO), 1% nucleosides (Millipore), 10% FBS (Millipore), β-mercaptoethanol (110 μM), and leukemia inhibitor factor (LIF, 10.000 units/mL; Millipore, ESG1106). Cells were maintained at 37 °C, 5% CO_2_, and regularly passaged using 0.5% trypsin (Millipore).

### Generation of mouse embryonic stem cell-derived motor neurons

The protocol for generating mESC-derived motor neurons was previously published (Wichterle *et al*., 2013). Briefly, mESC are trypsinized at confluency, counted and seeded at the concentration 100.000 to 200.000 cells/mL in low adherent cell culture dish in DFNK medium composed of advanced DMEM/F12 (44%), neurobasal medium (44%), KSR (10%), L-glutamine (2 mM), Penicillin-Streptomycin (100 Units/mL and 100 mg/mL, respectively) and β-mercaptoethanol (11 μM). The day after (=D2), culture medium was changed by gently spinning down (0.2 rcf) the cultures to collect the EBs, which were then placed back into the low-adherent cell culture flasks with fresh DFNK medium. On day 3, to induce spinal cord patterning and production of motor neurons, the medium was changed and replaced with DFNK medium supplemented with RA (1 μM) and SAG (0.5 μM). Two days later, the medium was once again changed with fresh DFNK containing RA plus SAG. On day 7, the EBs containing between 50–70% *Hb9::GFP*-positive motor neurons were ready for dissociation and plating for further experiments.

### Generation of mouse and human embryonic stem cell-derived astrocytes

The protocol for generating mESC-derived astrocytes (mESC-astrocytes) was previously published (Roybon *et al*., 2013). Phase 1: Generation of MN cultures, as described above. Phase 2: on day 7 of differentiation, EBs are mechanically dissociated and grown in neurosphere medium composed of DMEM/F12 (Thermo Fisher), 2% B27 supplement, 2 mM L-glutamine, 100 mM non-essential amino acids, P/S (100 Units/mL and 100 mg/mL, respectively) and 2 mg/mL heparin (Sigma-Aldrich), supplemented with FGF2 (20 ng/mL) and EGF (20 ng/mL) for 2 weeks. Neurospheres, which contain growing neural progenitors, were mechanically passaged on day 14 to generate secondary neurospheres. Phase 3: on day 21, neurospheres were dissociated into a single cell suspension using trypsin/EDTA (1X), filtered (cell strainer size 70 μM), and neural progenitors were seeded on culture vessels coated with poly-ornithine (100 μg/mL) and laminin (2 μg/mL) in neuropshere medium supplemented with 10% FBS; medium was changed twice per week. If confluency was reached on day 28, cultures were passaged. The presence of immature astrocytes was determined by immunocytochemistry on day 35 (Fig. 1B). Human ESC-derived astrocytes were generated as previously described^19,70^.

### mESC-derived astrocytes and motor neurons co-culture

In order to investigate the influence of treated astrocytes on MN survival, *Hb9::GFP* MNs were co-cultured with non-treated astrocytes, or astrocytes treated with FGF18, FGF2, FBS, BMP4, or CNTF. Briefly, mESC-derived astrocytes aged for 35 days *in vitro* were plated on poly-ornithine/laminin 96-well plate at a density of 15,000 cells per well in neurosphere medium. After 24 hours, medium was replaced with neurosphere medium containing FGF18, FGF2, FBS, BMP4 or CNTF. On day 6, mESC-derived *Hb9::GFP*-positive MNs were seeded on the astrocyte monolayer at density of 5,000 cells per well in DFNK medium. After 48 hours, live cultures were imaged using the plate Runner HD (Trophos). The total number of *Hb9::GFP* MNs and mean outgrowth per MN was quantified using Metamorph image analysis software (Molecular Devices), using the module Neurite Outgrowth.

### Total RNA extraction

Astrocyte cultures were washed twice with PBS and stored at −80 °C. On the day of isolation of RNA, cultures were thawed on ice and total RNA was extracted using RNeasy mini kit (Qiagen) according to the manufacturer’s recommendation. RNA concentration and quality were measured using Nanodrop and total RNA was stored at −80 °C.

### GeneChip microarray assay

Total RNA quality was checked using a bioanalyzer Agilent 2100 (Agilent). Sample preparation for microarray hybridization was carried out according to the Affymetrix GeneChip WT PLUS Reagent Kit User Manual (Affymetrix, Inc., Santa Clara, CA, USA). In brief, 200 ng of total RNA were used to generate double-stranded cDNA. 12 µg of subsequently synthesized cRNA was purified and reverse transcribed into sense-strand (ss) cDNA to which unnatural dUTP residues were incorporated. Purified ss cDNA was fragmented using a combination of uracil DNA glycosylase (UDG) and apurinic/apyrimidinic endonuclease 1 followed by a terminal labeling with biotin. 3.8 µg of fragmented and labeled ss cDNA were hybridized to Affymetrix Mouse Gene 2.1 ST Array Plates. For hybridization, washing, staining and scanning an Affymetrix GeneTitan system, controlled by the Affymetrix GeneChip Command Console software v4.2, was used. Sample processing was performed at an Affymetrix Service Provider and Core Facility KFB - Center of Excellence for Fluorescent Bioanalytics (Regensburg, Germany; www.kfb-regensburg.de).

### Microarray data analysis

Summarized probe set signals in log_2_ scale were calculated by using the RMA algorithm^71^ with the Affymetrix GeneChip Expression Console v1.4 Software. Bioconductor was used for the analysis of expression data. Unless specified, probe sets with a change ≥4.0-fold up or down were examined; analysis was performed using a student’s *t* test. Genes with p values lower than 0.05 were considered as significantly regulated. Volcano plots were drawn based on fold-change and p-values calculated for each gene. Gene Ontology term enrichment was performed with WebGestalt^72^ by using the entire dataset as background. The raw data are available in Supplementary Files and they can also be accessed by simple request to the corresponding author.

### Screening for inducers of astrocytic GLT1

Confluent monolayers of mESC-derived astrocytes aged D35 were used to screen for inducers of GLT1 expression. Astrocytes were seeded in poly-ornithine/laminin coated clear bottom 96-well microplates (Greiner Bio One), at the concentration 10,000 cells/well, in basic neurosphere medium. After 24 hours, wells were rinsed to remove dead cells and debris. Each condition was added manually to the wells using a P200 pipette, by replacing the basic neurosphere medium with basic neurosphere medium containing the compounds. The final volume per condition equalled 200 μL. After a 6-day period the cultures were fixed with 4% paraformaldehyde and stained using antibodies raised against GLT1, GFAP, and AQP4. Nuclei were stained with DAPI. Whole-well images of stained cultures were captured using the plate Runner HD (Trophos) and analyzed for total cell number (based on DAPI staining). Marker expression and intensity staining were measured using Metamorph (Cell Scoring Application Module; Molecular Devices) image analysis software. Each screen was performed using astrocytes generated from a new differentiation starting from mESC stage. Since both wildtype and *Hb9::GFP* mESC lines responded to FGF2 treatment by increasing GLT1 expression, we plotted the values of 4 independent experiments (n = 3 for the wildtype mESC line and n = 1 for the *Hb9::GFP* line) together on the same graph, as mean ± SEM. The list and concentrations of the compounds added for each condition is presented in the Table 1 below. Data are presented as fold changes to control condition (=no compound in the medium).

### Screening for inducers of GLT1 on motor neurons

Seven-day old *Hb9::GFP* motor neuron-containing EBs were dissociated and cells were seeded on poly-ornithine/laminin coated (100 μg/mL and 15 μg/mL, respectively) clear bottom 96-well microplates (Greiner Bio One) at the concentration of 3,333 cells/well in DFNK medium supplemented with 10 μM of kenpaullone (Tocris). After 24 hours, wells were rinsed to remove dead cells and debris. As for astrocyte cultures, each condition was added manually to the wells using a P200 pipette. The concentration of the compound was similar to that used to test GLT1 induction. All compounds were tested in DFNK medium. The final volume per condition equalled 200 μL per well. Each condition was tested in duplicate wells. *Hb9-GFP*-positive MN survival was measured two days after adding the compounds. Whole-well images of stained cultures were captured using the plate Runner HD (Trophos) and analyzed for total motor neuron number (based on *Hb9::GFP* natural fluorescence) using the neurite outgrowth module of the Metamorph image analysis software (Molecular Devices) with optimized parameters (neurons were counted positive when neurite outgrowth was at least two times the size of the cell body). Each screen was performed using *Hb9::GFP* motor neurons generated from a new differentiation starting from mESC stage. The screening was performed in duplicate and by two separate investigators. Values were plotted as mean ± SEM. Data were changed to represent fold change to control condition (control condition = medium with no added compound).

### Immunocytochemistry

ICC was carried out using standard protocols. The used antibodies were anti-GFAP (Rabbit polyclonal; 1:1,000; DAKO), anti-GFAP (mouse monoclonal; 1:1,000; Sigma); anti-AQP4 (Rabbit polyclonal; 1:400; Santa Cruz), and anti-GLT1 (Jeffrey Rothstein laboratory). The secondary antibodies ALEXA-FLUOR-488, ALEXA-FLUOR-555 and ALEXA-FLUOR-647 (Thermo Fisher Scientific) were used at the dilution 1:400; DAPI (1:50,000) was used to stain nuclei (Thermo Fisher Scientific).

### Western blotting

Briefly, protein concentrations were measured using Bradford Bio-Rad protein assay according to the manufacturer’s protocol (Bio-Rad, USA). The absorbance measurement was performed following manufacturer’s protocol (Biochrom Asys Expert 96 micro plate reader, Cambridge, UK). 10 μg of proteins were loaded on 4–20% Mini-Protean TGX Precast Gels (Bio-Rad, USA) then transferred to nitrocellulose membranes (Bio-Rad) using Trans-Blot Turbo System (Bio-Rad). Membranes were then blocked with 5% skim milk (Sigma-Aldrich) diluted in PBS-Tween 20 (Sigma-Aldrich). After blocking, the membranes were incubated with the primary antibodies listed in previous section and BMP7 antibody (Abcam; data not shown) at 4 °C, overnight. Incubation with primary monoclonal anti-ß-Actin antibody (1:10,000; Sigma-Aldrich) was performed for 20 minutes only. Membranes were incubated with peroxidase-conjugated secondary antibody (Vector Labs) and blots were developed using Clarity Western ECL Substrate (Bio-Rad) and the protein levels were normalized to actin.

### Image acquisition and statistical analysis

High magnification images were acquired using an inverted epifluorescence microscope LRI-Olympus IX-73 equipped with a Hamamatsu ORCA Flash camera. Per experiment, 3–4 random fields of view were counted using unbiased automated cell counting [Metamorph software, multi-wavelength cell scoring module (Molecular Devices)]. Images from up to 3 separate wells were analyzed. Sample groups were subjected to unpaired t-test. Whole-well images of stained astrocyte cultures and *Hb9::GFP*-positive MNs were captured using the Plate Runner HD (Trophos). For each experiment, duplicate wells were tested per condition. At least 3 independent experiments were performed (except for screening MN survival where 2 independent experiments were performed), and for each experiment, astrocytes and motor neurons were generated from mESC stage. The means of the sample groups were compared using one-or two-way ANOVA statistical test with Dunnett or Fisher-post hoc multiple comparisons test when all columns were compared to control condition. All quantitative data were analyzed using Prism 7 (GraphPad). The null hypothesis was rejected at 0.05 for all ANOVAs and post hoc tests. Figures were generated using Canvas Draw for mac (v. 1.0.1.; ACD systems).

## Supplementary information

Savchenko et al supplementary text

Table S1_Savchenko et al_Affymetrix Result_FGF2

Table S2_Savchenko et al_Affymetrix Result_RMA_FGF18

## References

[1] Allaman I, Belanger M, Magistretti PJ (2011). Astrocyte-neuron metabolic relationships: for better and for worse. Trends Neurosci.

[2] Attwell D (2010). Glial and neuronal control of brain blood flow. Nature.

[3] Choi HB (2012). Metabolic communication between astrocytes and neurons via bicarbonate-responsive soluble adenylyl cyclase. Neuron.

[4] Clarke LE, Barres BA (2013). Glia keep synapse distribution under wraps. Cell.

[5] Simard M, Nedergaard M (2004). The neurobiology of glia in the context of water and ion homeostasis. Neuroscience.

[6] Pekny M, Wilhelmsson U, Pekna M (2014). The dual role of astrocyte activation and reactive gliosis. Neurosci Lett.

[7] Anderson CM, Swanson RA (2000). Astrocyte glutamate transport: review of properties, regulation, and physiological functions. Glia.

[8] Kim K (2011). Role of excitatory amino acid transporter-2 (EAAT2) and glutamate in neurodegeneration: opportunities for developing novel therapeutics. J Cell Physiol.

[9] Rothstein JD (1996). Knockout of glutamate transporters reveals a major role for astroglial transport in excitotoxicity and clearance of glutamate. Neuron.

[10] Danbolt NC (2001). Glutamate uptake. Prog Neurobiol.

[11] Foran E, Trotti D (2009). Glutamate transporters and the excitotoxic path to motor neuron degeneration in amyotrophic lateral sclerosis. Antioxid Redox Signal.

[12] Behrens PF, Franz P, Woodman B, Lindenberg KS, Landwehrmeyer GB (2002). Impaired glutamate transport and glutamate-glutamine cycling: downstream effects of the Huntington mutation. Brain.

[13] Bristol LA, Rothstein JD (1996). Glutamate transporter gene expression in amyotrophic lateral sclerosis motor cortex. Ann Neurol.

[14] Fontana AC (2015). Current approaches to enhance glutamate transporter function and expression. J Neurochem.

[15] Lin CL (1998). Aberrant RNA processing in a neurodegenerative disease: the cause for absent EAAT2, a glutamate transporter, in amyotrophic lateral sclerosis. Neuron.

[16] Rothstein JD, Van Kammen M, Levey AI, Martin LJ, Kuncl RW (1995). Selective loss of glial glutamate transporter GLT-1 in amyotrophic lateral sclerosis. Ann Neurol.

[17] Rao PS (2015). Effects of ampicillin, cefazolin and cefoperazone treatments on GLT-1 expressions in the mesocorticolimbic system and ethanol intake in alcohol-preferring rats. Neuroscience.

[18] Soni N, Reddy BV, Kumar P (2014). GLT-1 transporter: an effective pharmacological target for various neurological disorders. Pharmacol Biochem Behav.

[19] Roybon L (2013). Human stem cell-derived spinal cord astrocytes with defined mature or reactive phenotypes. Cell Rep.

[20] Yang Y (2009). Presynaptic regulation of astroglial excitatory neurotransmitter transporter GLT1. Neuron.

[21] Irmady K, Zechel S, Unsicker K (2011). Fibroblast growth factor 2 regulates astrocyte differentiation in a region-specific manner in the hindbrain. Glia.

[22] Murphy M, Drago J, Bartlett PF (1990). Fibroblast growth factor stimulates the proliferation and differentiation of neural precursor cells *in vitro*. J Neurosci Res.

[23] Reuss B (2003). & von Bohlen und Halbach, O. Fibroblast growth factors and their receptors in the central nervous system. Cell Tissue Res.

[24] Noda M (2014). FGF-2 released from degenerating neurons exerts microglial-induced neuroprotection via FGFR3-ERK signaling pathway. J Neuroinflammation.

[25] Goetz R, Mohammadi M (2013). Exploring mechanisms of FGF signalling through the lens of structural biology. Nat Rev Mol Cell Biol.

[26] Ornitz DM, Itoh N (2015). The Fibroblast Growth Factor signaling pathway. Wiley Interdiscip Rev Dev Biol.

[27] Yun YR (2010). Fibroblast growth factors: biology, function, and application for tissue regeneration. J Tissue Eng.

[28] Zubilewicz A (2001). Two distinct signalling pathways are involved in FGF2-stimulated proliferation of choriocapillary endothelial cells: a comparative study with VEGF. Oncogene.

[29] Goldshmit Y (2014). Fgf2 improves functional recovery-decreasing gliosis and increasing radial glia and neural progenitor cells after spinal cord injury. Brain Behav.

[30] Lee M (2011). Acidic fibroblast growth factor (FGF) potentiates glial-mediated neurotoxicity by activating FGFR2 IIIb protein. J Biol Chem.

[31] Shao X (2017). FGF2 cooperates with IL-17 to promote autoimmune inflammation. Sci Rep.

[32] Liddelow SA (2017). Neurotoxic reactive astrocytes are induced by activated microglia. Nature.

[33] Wichterle H, Lieberam I, Porter JA, Jessell TM (2002). Directed differentiation of embryonic stem cells into motor neurons. Cell.

[34] Balazs R (1997). Metabotropic glutamate receptor mGluR5 in astrocytes: pharmacological properties and agonist regulation. J Neurochem.

[35] Hochstim C, Deneen B, Lukaszewicz A, Zhou Q, Anderson DJ (2008). Identification of positionally distinct astrocyte subtypes whose identities are specified by a homeodomain code. Cell.

[36] Ashburner M (2000). Gene ontology: tool for the unification of biology. The Gene Ontology Consortium. Nat Genet.

[37] Beenken A, Mohammadi M (2009). The FGF family: biology, pathophysiology and therapy. Nat Rev Drug Discov.

[38] Hanson MG, Shen S, Wiemelt AP, McMorris FA, Barres BA (1998). Cyclic AMP elevation is sufficient to promote the survival of spinal motor neurons *in vitro*. J Neurosci.

[39] Henderson CE (1996). Role of neurotrophic factors in neuronal development. Curr Opin Neurobiol.

[40] Lamas NJ (2014). Neurotrophic requirements of human motor neurons defined using amplified and purified stem cell-derived cultures. PLoS One.

[41] Lindholm P, Saarma M (2010). Novel CDNF/MANF family of neurotrophic factors. Dev Neurobiol.

[42] Maden M (2006). Retinoids and spinal cord development. J Neurobiol.

[43] Davies JE (2008). Transplanted astrocytes derived from BMP- or CNTF-treated glial-restricted precursors have opposite effects on recovery and allodynia after spinal cord injury. J Biol.

[44] Magistri M (2016). A comparative transcriptomic analysis of astrocytes differentiation from human neural progenitor cells. Eur J Neurosci.

[45] Barberi T (2003). Neural subtype specification of fertilization and nuclear transfer embryonic stem cells and application in parkinsonian mice. Nat Biotechnol.

[46] Bonaguidi MA (2005). LIF and BMP signaling generate separate and discrete types of GFAP-expressing cells. Development.

[47] Krencik R, Weick JP, Liu Y, Zhang ZJ, Zhang SC (2011). Specification of transplantable astroglial subtypes from human pluripotent stem cells. Nat Biotechnol.

[48] Oksanen Minna, Petersen Andrew J., Naumenko Nikolay, Puttonen Katja, Lehtonen Šárka, Gubert Olivé Max, Shakirzyanova Anastasia, Leskelä Stina, Sarajärvi Timo, Viitanen Matti, Rinne Juha O., Hiltunen Mikko, Haapasalo Annakaisa, Giniatullin Rashid, Tavi Pasi, Zhang Su-Chun, Kanninen Katja M., Hämäläinen Riikka H., Koistinaho Jari (2017). PSEN1 Mutant iPSC-Derived Model Reveals Severe Astrocyte Pathology in Alzheimer's Disease. Stem Cell Reports.

[49] Kusakawa Y, Mikawa S, Sato K (2017). BMP7 expression in the adult rat brain. IBRO Rep.

[50] Dharmarajan S (2014). Bone morphogenetic protein 7 regulates reactive gliosis in retinal astrocytes and Muller glia. Mol Vis.

[51] Fuller ML (2007). Bone morphogenetic proteins promote gliosis in demyelinating spinal cord lesions. Ann Neurol.

[52] Chen C, Bai GC, Jin HL, Lei K, Li KX (2018). Local injection of bone morphogenetic protein 7 promotes neuronal regeneration and motor function recovery after acute spinal cord injury. Neural Regen Res.

[53] Setoguchi T (2001). Traumatic injury-induced BMP7 expression in the adult rat spinal cord. Brain Res.

[54] Cahoy JD (2008). A transcriptome database for astrocytes, neurons, and oligodendrocytes: a new resource for understanding brain development and function. J Neurosci.

[55] Dvorak P, Dvorakova D, Hampl A (2006). Fibroblast growth factor signaling in embryonic and cancer stem cells. FEBS Lett.

[56] Rai KS, Hattiangady B, Shetty AK (2007). Enhanced production and dendritic growth of new dentate granule cells in the middle-aged hippocampus following intracerebroventricular FGF-2 infusions. Eur J Neurosci.

[57] Scholze AR, Foo LC, Mulinyawe S, Barres BA (2014). BMP signaling in astrocytes downregulates EGFR to modulate survival and maturation. PLoS One.

[58] Krupenko SA (2009). FDH: an aldehyde dehydrogenase fusion enzyme in folate metabolism. Chem Biol Interact.

[59] Yang Y (2011). Molecular comparison of GLT1+ and ALDH1L1+ astrocytes *in vivo* in astroglial reporter mice. Glia.

[60] Brunne B (2010). Origin, maturation, and astroglial transformation of secondary radial glial cells in the developing dentate gyrus. Glia.

[61] Zhang X (2006). Receptor specificity of the fibroblast growth factor family. The complete mammalian FGF family. J Biol Chem.

[62] Choubey L, Collette JC, Smith KM (2017). Quantitative assessment of fibroblast growth factor receptor 1 expression in neurons and glia. PeerJ.

[63] Dombrowski C (2013). FGFR1 signaling stimulates proliferation of human mesenchymal stem cells by inhibiting the cyclin-dependent kinase inhibitors p21(Waf1) and p27(Kip1). Stem Cells.

[64] Grabiec M (2016). Stage-specific roles of FGF2 signaling in human neural development. Stem Cell Res.

[65] Zhang J, Upadhya D, Lu L, Reneker LW (2015). Fibroblast growth factor receptor 2 (FGFR2) is required for corneal epithelial cell proliferation and differentiation during embryonic development. PLoS One.

[66] Ito K (2018). Lack of Fgf18 causes abnormal clustering of motor nerve terminals at the neuromuscular junction with reduced acetylcholine receptor clusters. Sci Rep.

[67] Dezonne RS (2017). Derivation of Functional Human Astrocytes from Cerebral Organoids. Sci Rep.

[68] Santos R (2017). Differentiation of Inflammation-Responsive Astrocytes from Glial Progenitors Generated from Human Induced Pluripotent Stem Cells. Stem Cell Reports.

[69] Nakada M (2011). Aberrant signaling pathways in glioma. Cancers (Basel).

[70] Holmqvist S (2015). Generation of human pluripotent stem cell reporter lines for the isolation of and reporting on astrocytes generated from ventral midbrain and ventral spinal cord neural progenitors. Stem Cell Res.

[71] Irizarry RA (2003). Exploration, normalization, and summaries of high density oligonucleotide array probe level data. Biostatistics.

[72] Wang J, Vasaikar S, Shi Z, Greer M, Zhang B (2017). WebGestalt 2017: a more comprehensive, powerful, flexible and interactive gene set enrichment analysis toolkit. Nucleic Acids Res.

