# Supplementary material for: FGF family members differentially regulate maturation and proliferation of stem cell-derived astrocytes
Source: Sci Rep. 2019 Jul 3;9:9610. doi: 10.1038/s41598-019-46110-1 (PMC6610107; doi:10.1038/s41598-019-46110-1)
Supplement: Supplementary file 1 — Savchenko et al supplementary text [file 41598_2019_46110_MOESM1_ESM.docx]

**SUPPLEMENTARY INFORMATION TO ARTICLE**

**TITLE**

FGF family members differentially regulate maturation and proliferation of stem cell-derived astrocytes

**AUTHORS**

Ekaterina Savchenko^1^, Gabriel N. Teku^2^, Antonio Boza-Serrano^3^, Kaspar Russ^1^, Manon Berns^1^, Tomas Deierborg^3^, Nuno J. Lamas^4,5^, Hynek Wichterle^6^, Jeffrey Rothstein^7^, Christopher E. Henderson^8^, Mauno Vihinen^2^, Laurent Roybon^1^*

**
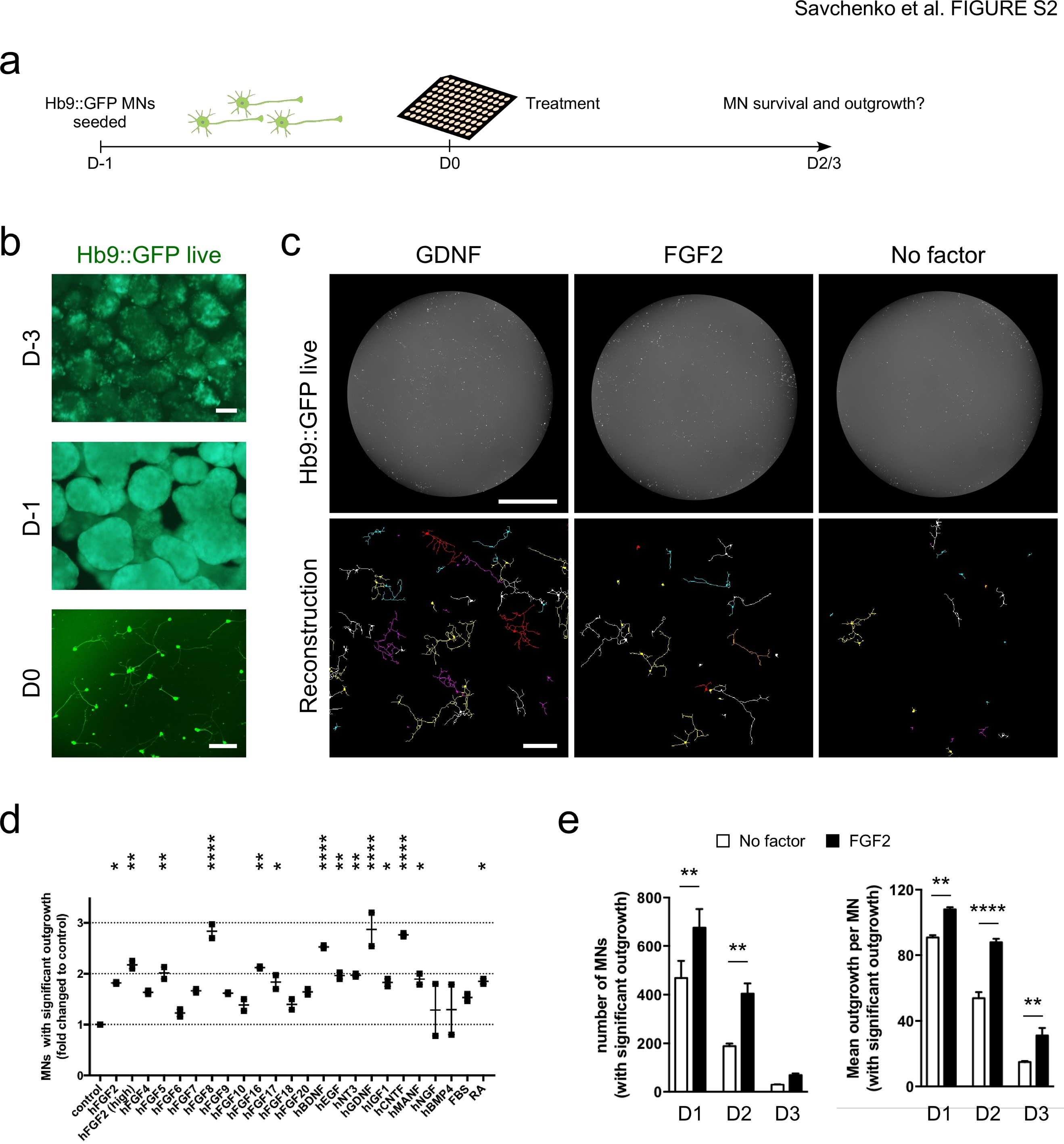
**

***Figure S1: Effect of factors on MN survival and outgrowth***

**A-** Overview of the screening flow employed to examine MN survival and neurite outgrowth. **B-** Representative images of *Hb9::GFP* MN cultures during differentiation, 3 days prior to dissociation (upper panel), on the day of dissociation (middle panel), and a day after seeding prior to adding the conditions (lower panel). Scale bars = 100 μm (upper panel) and 75 μm (lower panel).

**C-** Whole well image depicting live *Hb9::GFP* MNs 2 days after treatment. Representative images are showed for cultures treated with GDNF (50 ng/mL, positive control) and FGF2 (50 ng/mL), and non-treated (negative control). Lower panels show images reconstructed using metamorph software, module neurite outgrowth. Scale bars = 200 μm (upper panel) and 100 μm (lower panel).

**D-** Quantification of the number of *Hb9::GFP* MNs in culture aged 2 days *in vitro* following treatment with the factors we tested on astrocyte cultures. Data are presented as fold change to control. Mean ± SEM; treatment performed for n = 2 independent differentiations, with duplicate wells examined for each screen. *P* values: * = P<0.05; ** = P<0.01; *** = P<0.001; **** = P<0.0001.

**E-** Bar diagrams represent the total number of *Hb9::GFP* MNs (left) and the mean outgrowth per MN (right) for cultures treated with FGF2 and non-treated, over a time course of 3 days. Mean ± SEM; n = 3 independent differentiations. *P* values: * = P<0.05; ** = P<0.01; *** = P<0.001; **** = P<0.0001.

**
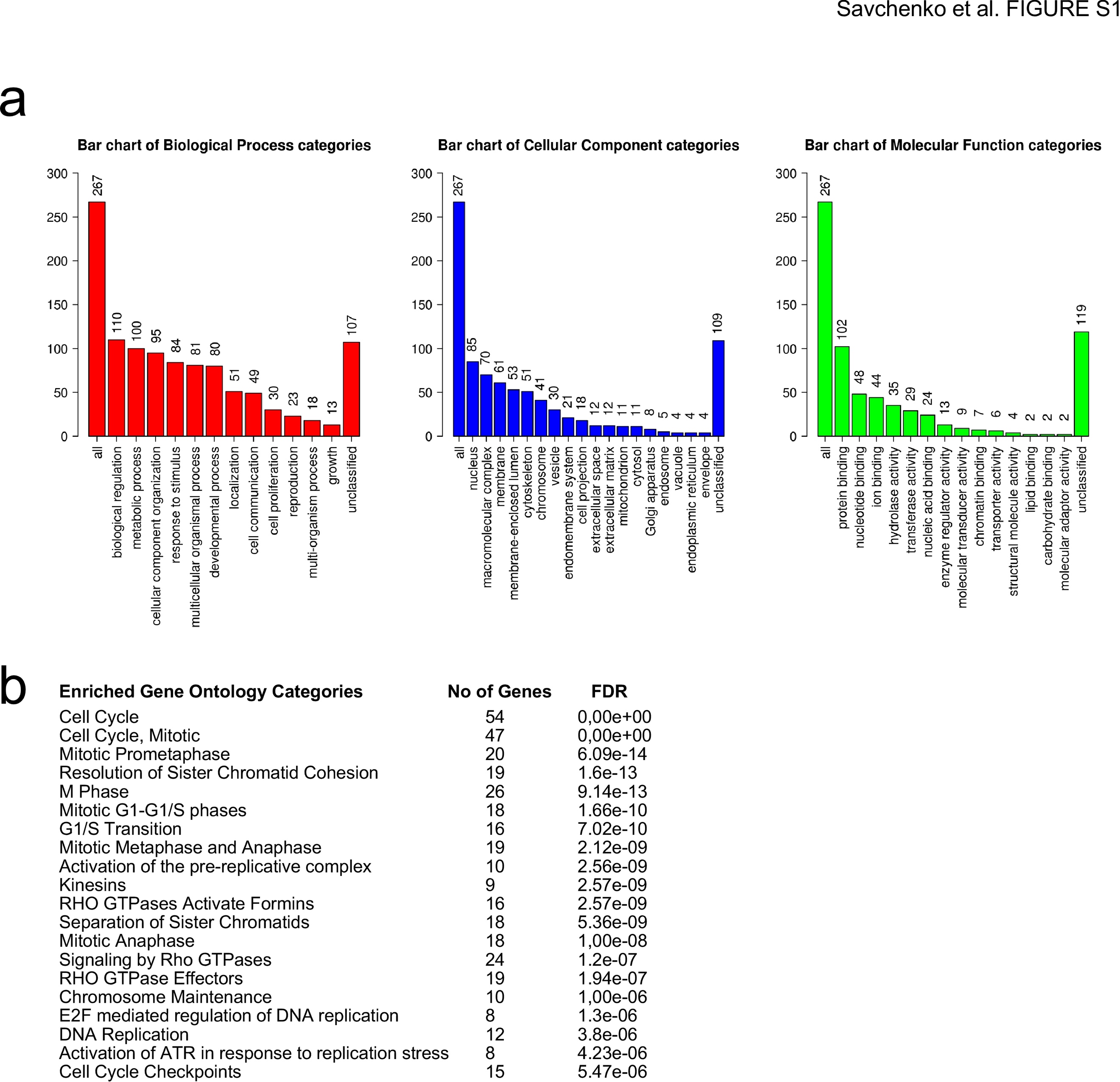
**

***Figure S2: Analysis of the astrocyte cultures transcriptome prior to and after FGF2 treatment.***

**A-** Enriched GO Slim terms for biological process, cellular compartment and molecular function categories for genes with at least 4-fold up or down regulation. 268 genes were unambiguously mapped to unique Entrez gene identifiers.

**B-** Significantly enriched GO Slim terms in the Reactome pathways, for genes with over four-fold expression change. 268 genes were unambiguously mapped to unique Entrez gene identifiers


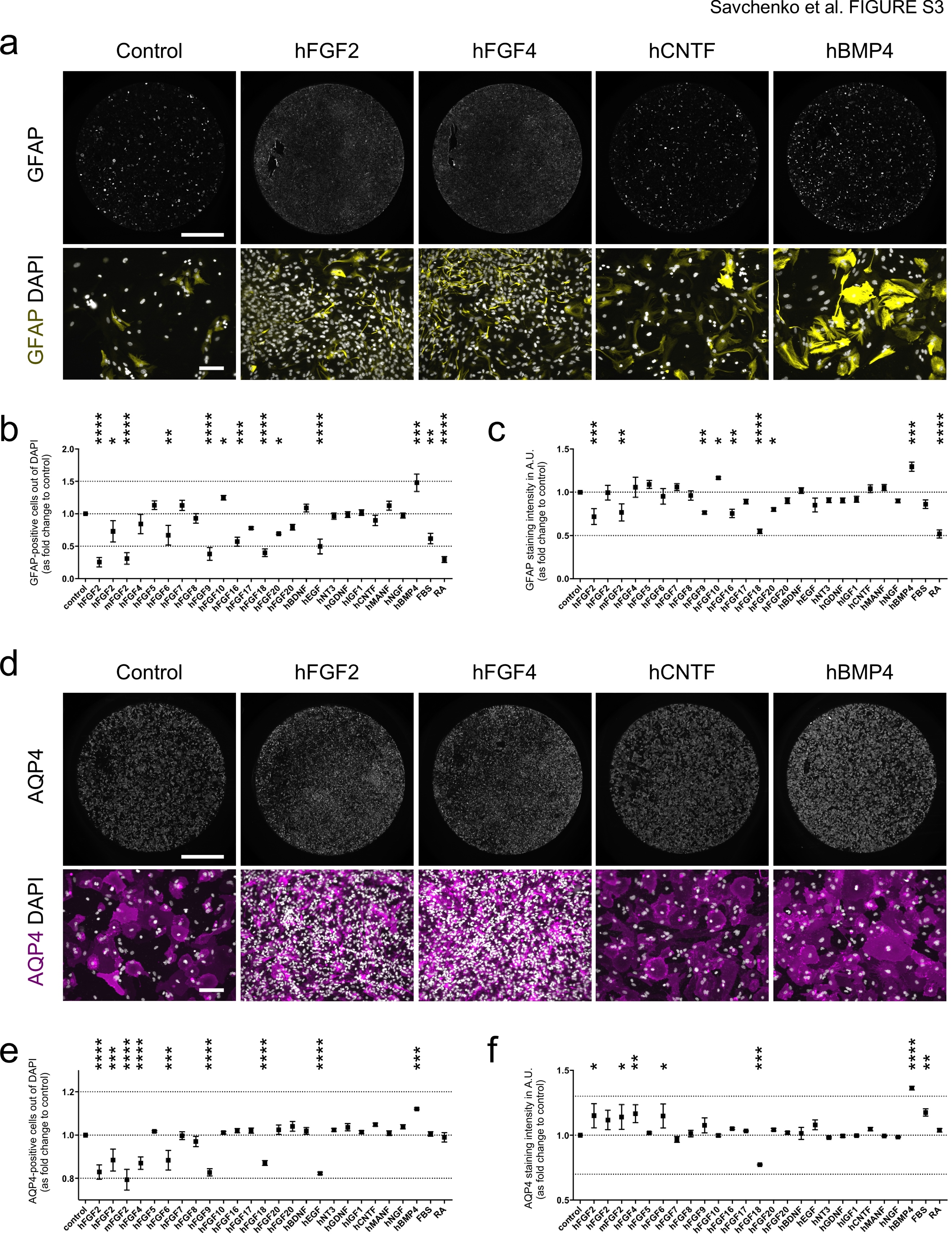


***Figure S3: Characterization of astrocyte cultures treated with the factors.***

**A-** Whole well image depicting GFAP immunofluorescence following immunocytochemistry. Representative images from one of the 4 screens are presented. Cultures were treated for 6 days with different conditions without media change. Lower panels show high magnification images of non-treated, FGF2, FGF4, CNTF and BMP4 treated astrocyte cultures, stained for GFAP and DAPI (images are representative of n = 4 independent experiments). Scale bars = 200 μm (upper panel) 75 μm (lower panel).

**B-** Bar diagram represents the proportion of GFAP-positive cells in each culture condition (data are presented as fold-change of the non-treated cultures). Mean ± SEM; treatment performed for n= 4 independent differentiations. *P* values: * = P<0.05; ** = P>0.01; *** = P<0.001; **** = P<0.0001.

**C-** Bar diagram represents the average intensity staining per cell in each culture condition (data are presented as fold-change of the non-treated condition). Mean ± SEM; n= 4 independent differentiations. *P* values: * = P<0.05; ** = P>0.01; *** = P<0.001; **** = P<0.0001.

**D-** Whole well image depicting AQP4 immunofluorescence following immunocytochemistry. Representative images from one of the 4 screens are presented. Cultures were treated for 6 days with the conditions, with no media change. Lower panels show high magnification images of non-treated, FGF2, FGF4, CNTF and BMP4 treated astrocyte cultures, stained for AQP4 and DAPI (images are representative of n = 4 independent experiments). Scale bars = 200 μm (upper panel) 75 μm (lower panel).

**E-** Bar diagram represents the proportion of AQP4-positive cells in each culture condition (data are presented as fold-change of the non-treated cultures). Mean ± SEM; treatment performed for n= 4 independent differentiations. *P* values: * = P<0.05; ** = P>0.01; *** = P<0.001; **** = P<0.0001.

**F-** Bar diagram represents the average intensity staining per cell in each culture condition (data are presented as fold-change of the non-treated condition). Mean ± SEM; n= 4 independent differentiations. *P* values: * = P<0.05; ** = P>0.01; *** = P<0.001; **** = P<0.0001.


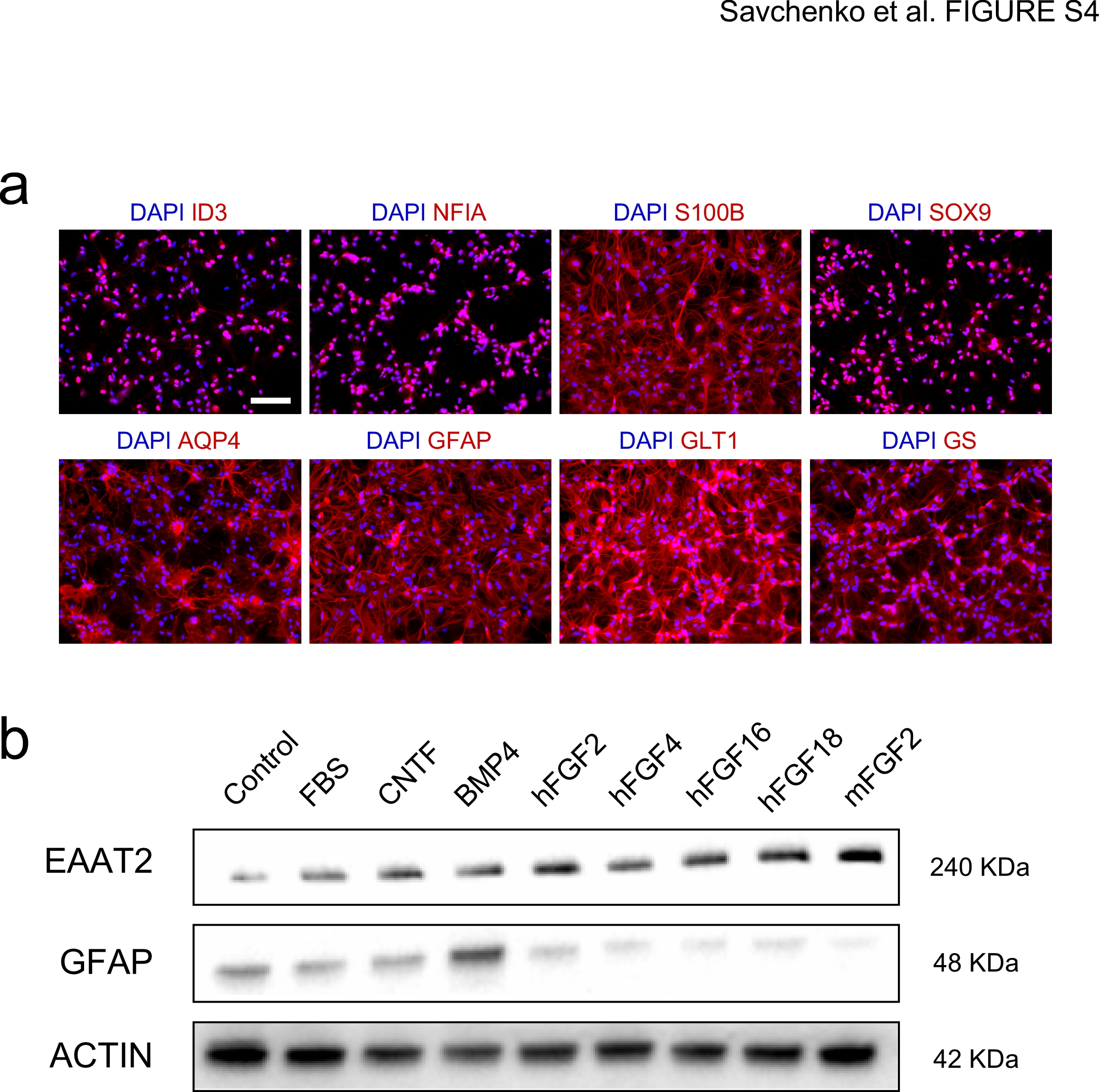


***Figure S4: Effect of FBS, CNTF, BMP4, FGF2, FGF4, FGF16, FGF18 on human ESC-derived astrocytes.***

**A-** Human astrocytes generated from human ESC (Holmqvist et al, 2015) express canonical markers ID3, NFIA, S100b, SOX9, AQP4, GFAP, EAAT2 and GS. Scale bar = 75 μm.

**B-** Representative Western blot of hESC-derived astrocyte cultures following treatment with FBS, CNTF, BMP4, FGF2, FGF4, FGF16, FGF18. GLT1 is increased in cultures treated for 6 days with the FGFs, compared to non-treated, FBS-, CNTF- and BMP4-treated astrocyte cultures of the same age. GFAP is decreased in cultures treated for 6 days with the FGFs, compared to non-treated, FBS-, CNTF- and BMP4-treated astrocyte cultures of the same age. Actin protein was used as loading control (blots are representative of two independent experiments).
